# S48. Biomarker development for ipilimumab and prostate GVAX treatment

**DOI:** 10.1186/2051-1426-2-S2-I11

**Published:** 2014-03-12

**Authors:** T de Gruijl, S Santegoets, A Stam, S Lougheed, K Jooss, N Sacks, T Harding, K Hege, W Gerritsen, A van den Eertwegh

**Affiliations:** 1 VU University Medical Center, Medical Oncology, Amsterdam, the Netherlands; 2 Cell Genesys Inc., South San Francisco, CA, USA

## Background

Immunotherapeutic approaches such as vaccination or immune checkpoint blockade have proven to be clinically active in prostate cancer, but only in fractions of treated patients; this calls for personalized application of these novel therapies based on predictive biomarkers.

## Methods

Our own research over the past years has focused on the clinical efficacy in patients with castration-resistant prostate cancer of the combination of an allogeneic cell line-based vaccine (Prostate GVAX) and an anti-CTLA4 checkpoint inhibitor (ipilimumab) in a Phase-I/II dose escalation/expansion trial. We carried out an extensive immune monitoring programme comprising flowcytometric profiling of lymphoid and myeloid subsets in peripheral blood (PB) and T cell and serological reactivity to a panel of known tumor antigens, all before and after treatment.

## Results

On-treatment PSA declines of more than 50% were observed in 5, and PSA stabilizations in 12 of 28 patients. Regressing bone and lymph node metastases were observed in 2/5 responding patients. Significantly prolonged overall survival (OS) was observed for patients with high pre-treatment frequencies of CD4+CTLA-4+, CD4+PD-1+, or differentiated CD8+ T cells, or low pre-treatment frequencies of regulatory T cells. Treatment-induced activation of PB Dendritic Cell subsets was similarly associated with significantly prolonged OS. In contrast, high pre-treatment frequencies of monocytic Myeloid-Derived Suppressor Cells (MDSC) were associated with reduced OS. Th2/Th17 cytokine profiles were induced. Indeed, profound up-regulation of CD4+IL-5+ T cell frequencies was associated with improved OS (p=0.03) and correlated significantly with the breadth of the induced antibody response. IgG antibody responses against 11 (prostate) tumor-associated antigens were determined and increased seroreactivity to prostate-specific membrane antigen (PSMA), pyridoxamine 5'-phosphate oxidase (PNPO) and/or Neuropilin-2 (NRP2) was significantly correlated with improved OS (p=0.002 for combined upregulated seroreactivity to all three). Finally, patients with pre-existing NY-ESO-1 T cell reactivity also demonstrated a significantly prolonged OS (p=0.044).

## Conclusion

Together these data provide an immune profile to predict clinical outcome. Importantly, cluster analysis revealed pre-treatment expression of CTLA-4 by circulating CD4+ T cells and an immune-stimulatory myeloid profile to be dominant predictors for OS after Prostate GVAX/ipilimumab therapy. These flowcytometry-based parameters may thus provide potentially useful and easy-to-use biomarkers for patient selection.

